# Global burden of colorectal cancer from 1990 to 2021: a systematic analysis from the Global Burden of Disease Study 2021

**DOI:** 10.3389/fonc.2025.1676855

**Published:** 2025-12-16

**Authors:** Xue Chen, Rui Tian, Ze Chen, Longfang Quan, Shaosheng Bei

**Affiliations:** Department of Proctology, Xiyuan Hospital, China Academy of Chinese Medical Sciences, Beijing, China

**Keywords:** colorectal cancer, disability-adjusted life years, Global Burden of Disease study, incidence, mortality, temporal trends

## Abstract

**Background:**

Colorectal cancer (CRC), currently the second leading cause of cancer-related mortality worldwide, poses a significant burden on public health. This study systematically analyzes the temporal trends in CRC disease burden based on Global Burden of Disease (GBD) data from 1990 to 2021, aiming to provide robust evidence for epidemiological research, disease prevention, and the formulation of public health policies.

**Methods:**

This study analyzed CRC incidence, mortality, and disability-adjusted life years (DALYs) using GBD1990–2021 data. Temporal trends were evaluated via estimated annual percentage changes (EAPC), with Pearson correlation assessing Socio-demographic Index (SDI) associations. Projections of global CRC epidemiology through 2035 were developed to inform public health strategies.

**Results:**

In 2021, global CRC accounted for 2,194,143 incident cases, 1,044,072 deaths, and 24,401,100 DALYs. CRC remains a major public health challenge worldwide. From 1990 to 2021, age-standardized incidence rates (ASIR) increased (EAPC = 0.15, 95% CI: 0.12–0.19), while mortality (EAPC=−0.81, −0.84 to −0.77) and DALY rates (EAPC=−0.83, −0.87 to −0.80) declined significantly. Notable socioeconomic gradients were observed across the spectrum of regions. Geographic disparities were prominent: high-SDI regions had the highest ASIR (40.525, 37.445–42.447 per 100,000), whereas high-middle-SDI regions showed peak mortality (15.709, 14.144–17.25) and DALY rates (338.225, 316.751–354.913). Males and individuals aged >85 years experienced disproportionate burden increases. By 2035, the global burden of CRC is projected to maintain its current upward trajectory.

**Conclusions:**

Globally, CRC’s ASIR has gradually increased, while age-standardized death rate (ASDR) and DALY rates have declined significantly, reflecting an overall reduction in disease burden. Regions with higher SDI, male predominance, and aging populations contribute most to rising CRC cases. Despite progress in mortality reduction, CRC prevention and control will continue to pose significant public health challenges in the coming years.

## Introduction

Colorectal cancer (CRC) is a common malignant tumor of the digestive tract, typically progressing from colonic or rectal adenomas. According to the 10th Revision of the International Classification of Diseases (ICD-10, 24th Edition), CRC is coded as C18–C21, D01.0–D01.2, or D12–D12.91 (1). Approximately 30–40% of CRC patients develop metastases during the course of the disease, and the vast majority of metastatic CRC (mCRC) cases are considered incurable (2). Studies indicate that CRC is strongly associated with multiple risk factors, such as a diet high in red and processed meats, lack of exercise, smoking, and alcohol use (3). The heterogeneous distribution of these risks among regions and populations markedly influences the disease’s epidemiological profile. According to estimates from the 2019 Global Burden of Disease (GBD) study, there were 2.17 million new CRC cases globally, resulting in 24.3 million disability-adjusted life years (DALYs) (95% uncertainty interval [UI]: 22.6–25.7 million), ranking first among digestive system cancers (4, 5). As a debilitating disease, CRC can lead to various complications, including anorexia, nausea, vomiting, early satiety, pain, bleeding, and bowel obstruction. These complications significantly shorten patients’ lifespans and diminish their quality of life (6). It also imposes a substantial burden on global healthcare systems.

Epidemiological studies show that the approximately 20-fold global variation in CRC incidence, with over half of cases concentrated in high-incidence regions like North America and Western Europe, is largely attributable to disparities in lifestyle, dietary habits, and the distribution of healthcare resources across regions and countries (7–9). In addition, the COVID-19 pandemic has had a significant impact on CRC screening, diagnosis, and treatment, further exacerbating the global disease burden of CRC (10). A comprehensive understanding of the changes and trends in the global disease burden of CRC in recent years requires a global perspective. By analyzing health data segmented by population, socioeconomic, or geographical factors, it is possible to reveal inequalities among different subgroups. This approach allows for the adjustment of policies, optimization of planning, and implementation of targeted interventions, aiming to establish a tiered healthcare service structure and ultimately improve the health of vulnerable populations (11).

While prior GBD research has multidimensionally characterized the CRC burden, a consolidated analysis of recent trends, age-stratified sex differences, and future projections is often absent. To address this gap, our study leverages the most recent GBD 2021 data to conduct a systematic analysis of global, regional, and national trends in CRC incidence, mortality, and DALYs from 1990 to 2021. By comparing burden distributions across sex, age, and SDI groups, this work aims to deliver a more robust evidence base for shaping CRC prevention and public health policies.

## Methods

### Data source

The CRC data analyzed in this study were sourced from GBD 2021. All data were systematically integrated and cataloged through the Global Health Data Exchange (GHDx) platform, which maintains comprehensive metadata including citation details, data providers, and access information. The official methodology employs data models to handle missing values, conducts uncertainty distribution estimations for all numerical outputs, and reports 95% uncertainty intervals to fully demonstrate the strength of evidence for each data point. This research framework aims to provide a comprehensive assessment of health losses attributable to 371 diseases and injuries across 204 countries and territories worldwide.

In GBD 2021, causes are categorized into four levels, ranging from level 1 for non-communicable diseases to level 4 for benign and *in situ* colorectal tumors. CRC is classified as a level 3 cause in GBD 2021. This study integrates the data from GBD 2021 to assess the long-term trends in CRC across all age groups. Specifically, we extracted data on CRC incidence, mortality, and DALYs, along with corresponding age-standardized rates (ASR), including age-standardized incidence rate (ASIR), age-standardized death rate (ASDR), and age-standardized DALYs rate from 1990 to 2021 by age, gender, region, country, and The Socio-demographic Index(SDI) through the Global Health Data Exchange platform (https://www.healthdata.org/research-analysis/gbd) (12, 13).The data for this study were collected and accessed on November 24, 2024. As the study utilized publicly available databases, it did not require ethical approval or informed consent.

### SDI

The SDI is a composite indicator introduced by the Institute for Health Metrics and Evaluation (IHME) in 2015. It aims to assess the development level of a country or region, emphasizing the interconnection between social development and population health outcomes (14). Socio-demographic development has been a major contributor to global health gains over the past 30 years. The SDI is a composite indicator representing the geometric mean of three parameters: lag-distributed income per capita, average years of education, and the fertility rate among females under 25 years in a specific location. The SDI score ranges from 0 (lowest income and education levels, highest fertility rate) to 100 (highest income and education levels, lowest fertility rate) (13). GBD 2021 categorizes 204 countries and regions into five groups based on their SDI levels: low (< 0.47), low-middle (0.47–0.62), middle (0.63–0.71), high-middle (0.72–0.81), and high (> 0.81) (13).

### Statistical analysis

#### Descriptive analysis

This study employed a descriptive analysis to examine the temporal and age-specific trends of CRC disease burden at the global, regional, and national levels. To account for demographic differences, ASIR, ASDR, and age-standardized DALYs rate were used to better reflect the actual patterns of incidence and mortality. All rates are expressed per 100,000 population.

### Estimated annual percentage change (EAPC)

EAPC is an effective and widely used metric that has been extensively applied in previous studies to track trends in indicators such as prevalence and incidence over a specific period. EAPC is calculated using a linear regression model with the equation:


$$\begin{array}{l} \text{y}\;=\;\text{α}\;+\;\beta \text{x}\;+\;\text{ϵ}\\ \text{x}-\;\text{year},\\ \text{y}-\;\text{the}\;\mathrm{natural}\;\mathrm{logarithm}\;\text{of}\;\text{the}\;\text{rate}\;\left(\text{e}.\text{g}.,\;\mathrm{prevalence}\;\mathrm{or}\;\mathrm{incidence}\right),\\ \alpha -\;\mathrm{the}\;\mathrm{intercept},\\ \beta -\;\text{the}\;\mathrm{slope},\\ \epsilon -\;\text{the}\;\text{random}\;\text{error}.\\ \text{EAPC}=100\times \left[\exp\left(\beta \right)-1\right] \end{array}$$

This approach provides a quantitative measure of the trend’s direction and magnitude (14). An EAPC and its 95% confidence interval (CI) greater than 0 indicate an increasing trend over time, whereas values less than 0 suggest a decreasing trend. If the 95% CI includes 0, it implies that the trend change is not statistically significant.

### Bayesian age-period-cohort model

To further predict the burden of CRC from 2021 to 2035, we obtained global population estimates for the period 2017–2100 from the GBD database (https://ghdx.healthdata.org/record/ihme-data/global-population-forecasts-2017-2100). We used the BAPC model with nested Laplace approximation, based on the assumption of a relationship between incidence or mortality and age structure and population size, for prediction analysis (15). The model captures disease burden trends across different time periods and populations by integrating age, period, and cohort effects. It is based on Bayesian statistical inference, utilizing historical data to predict future disease burden. In this model architecture, all unknown parameters are defined as random variables with specific prior distributions. Specifically, the intercept and linear trend terms are assigned vague Gaussian priors. For the random effects representing nonlinear variations in age, period, and birth cohort, intrinsic conditional autoregressive priors are employed to achieve smoothing across adjacent groups. The convergence of the Markov chain Monte Carlo algorithm was assessed using the Gelman-Rubin diagnostic (potential scale reduction factor, R-hat< 1.05). In the systematic comparison with the generalized Lee–Carter (LC) model, the BAPC model demonstrated lower Continuous Ranked Probability Score (CRPS) and Absolute Error (AE) across all countries, indicating superior predictive accuracy (16).

The advantage of the BAPC model is that it directly approximates the posterior marginal distribution, does not require convergence diagnostics, and has sufficient accuracy. Compared with the generalized additive model, the smooth spline model, the Nordpred model, and Poisson regression, the BAPC model has higher accuracy in predicting the short- and medium-term burden (15).

The statistical analysis for this study was conducted in R 4.3.2 (R Foundation for Statistical Computing, Vienna, Austria), with a p-value of less than 0.05 considered statistically significant. For visualization, we utilized the ggplot2 and maps packages in R to generate spatial distribution maps and temporal trends of the global CRC burden. The combined use of these tools provided strong support for data processing, analysis, and presentation, ensuring the accuracy of the analysis and clarity in the visualization.

## Result

### Overview of the global burden

**Figure 1:**
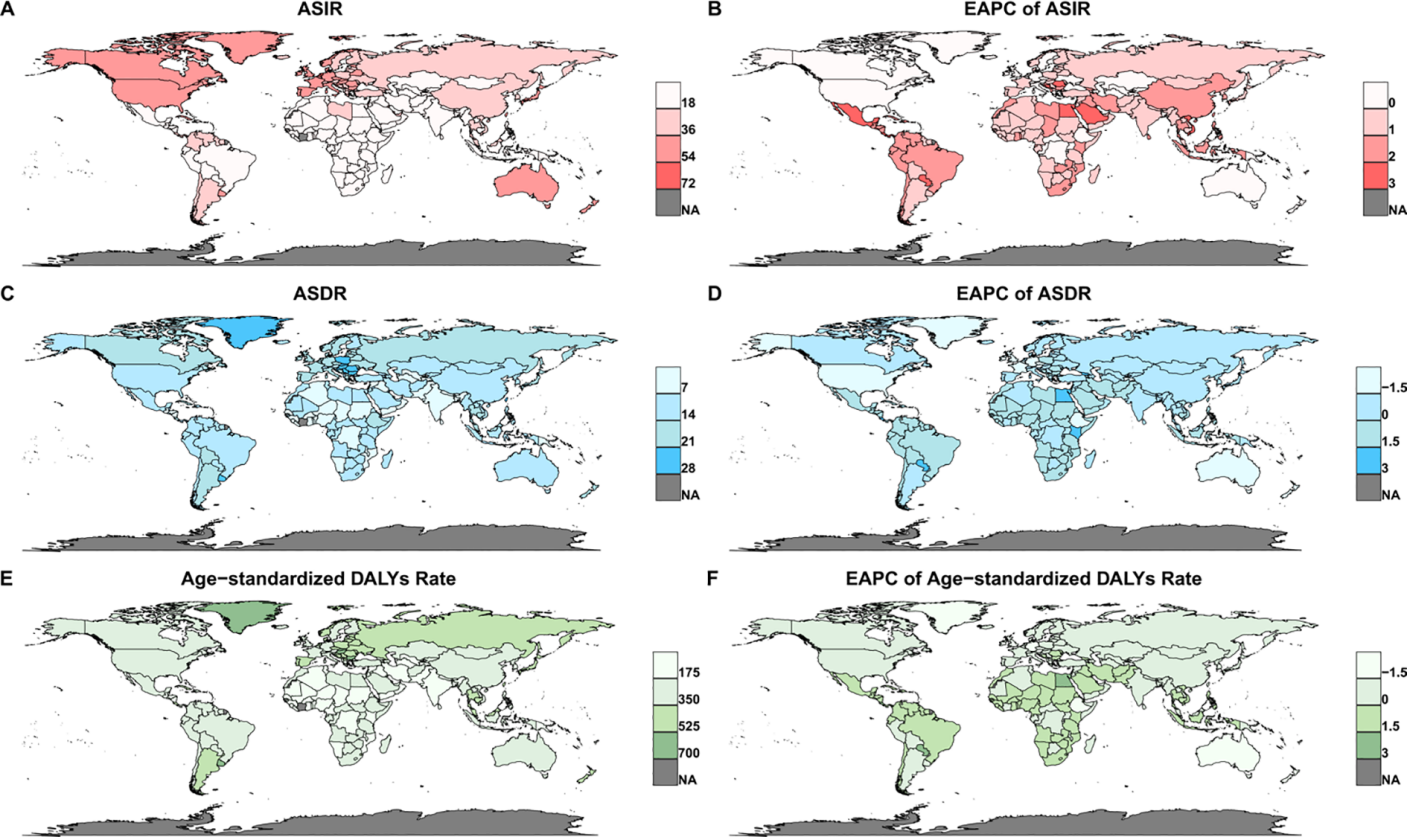
The ASIR **(A)**, ASDR **(C)**, Age-standardized DALYs rate **(E)**, and their EAPCs **(B, D, F)** of CRC across 204 countries and territories from 1990 to 2021. ASIR, Age-Standardized Incidence Rate, ASDR, Age-Standardized Death Rate; DALYs, Disability-Adjusted Life Years; EAPC, Estimated Annual Percentage Change; CRC, Colorectal Cancer.

We first conducted a statistical analysis of the global CRC disease burden and incidence trends. **Figure 1** and **Table 1** graphically depict the geographic distribution of the CRC burden across different regions worldwide. In 2021, the global numbers of CRC cases, deaths, and DALYs were 2,194,143 (95% UI: 2,001,272–2,359,390), 1,044,072 (95% UI: 950,188–1,120,169), and 24,401,100 (95% UI: 22,689,369–26,161,518), respectively (**Tables 1**, **2**, **Figure 1**). In 2021, the global ASIR was 25.607 (95% UI: 23.322–27.516), with an EAPC of 0.15 (95% CI: 0.12–0.19). Among regions, the highest ASIR was observed in high-income Asia Pacific populations at 44.89 (95% UI: 40.2–47.85), followed by Australia at 43.968 (95% UI: 38.907–49.552) and Western Europe at 40.54 (95% UI: 37.17–43.067). At the national level, the Netherlands recorded the highest overall ASIR of 69.803 (95% UI: 62.211–76.792), followed by Morocco at 68.334 (95% UI: 54.046–83.19) and Bermuda at 61.786 (95% UI: 51.462–77.112) (**Table 1**, **Figure 1**).

**Table 1:** The incidence, deaths, DALYs, ASIR, ASDR, and EAPCs of CRC at the global, regional levels, categorized by SDI and sex from 1990 to 2021.

Characteristics	1990			2021			1990			2021			
	Incident cases	ASIR		Incident cases	ASIR	EAPC	Death cases	ASDR		Death cases		ASDR	EAPC
	(95%UI)	per 100,000(95%UI)		(95%UI)	per 100,000(95%UI)	per 100,000(95%CI)	(95%UI)	per 100,000(95%UI)		(95%UI)		per 100,000(95%UI)	per 100,000(95%CI)
Global	916584 (866238-951895)	24.04 (22.544-25.011)		2194143 (2001272-2359390)	25.607 (23.322-27.516)	0.15 (0.12 - 0.19)	570319 (536545-597669)	15.562 (14.487-16.313)		1044072 (950188-1120169)		12.398 (11.241-13.306)	-0.81 (-0.84 to -0.77)
Gender													
Female	446821 (414136-472553)	21.41 (19.748-22.623)		930681 (824674-1017652)	20.171 (17.86-22.046)	-0.29 (-0.34 to -0.25)	282608 (258886-301416)	13.888 (12.681-14.804)		462515 (407296-503539)		9.963 (8.784-10.843)	-1.19 (-1.24 to -1.14)
Male	469763 (445308-492302)	27.306 (25.889-28.513)		1263462 (1146499-1400377)	31.928 (29.038-35.256)	0.50 (0.45 - 0.54)	287711 (269819-304982)	17.719 (16.665-18.675)		581557 (528253-641420)		15.346 (13.944-16.871)	-0.50 (-0.52 to -0.47)
SDI regions													
Low SDI	16472 (12928-18871)	7.319 (5.82-8.357)		36649 (32707-40883)	7.391 (6.652-8.187)	-0.05 (-0.21 - 0.11)	15462 (12106-17687)	7.214 (5.745-8.208)		31848 (28504-35514)		6.877 (6.181-7.615)	-0.21 (-0.39 to -0.04)
Low-middle SDI	38286 (33160-43284)	6.151 (5.365-6.935)		119416 (109344-130919)	8.195 (7.512-8.963)	0.96 (0.91 - 1.02)	33887 (29282-38356)	5.722 (4.992-6.449)		91274 (83733-99854)		6.552 (6.021-7.17)	0.43 (0.48 - 0.54)
Middle SDI	134432 (121315-148566)	12.897 (11.649-14.157)		526190 (462016-595124)	19.547 (17.141-22.043)	1.33 (0.96 - 1.70)	105725 (95513-116511)	10.795 (9.76-11.817)		274871 (243369-306694)		10.645 (9.424-11.844)	0.48 (0.43 - 0.54)
High-middle SDI	250999 (237456-263213)	25.583 (24.124-26.807)		669660 (598306-746397)	34.002 (30.33-37.95)	0.86 (0.44 - 1.29)	171222 (160927-179670)	18.124 (17.02-19.012)		308175 (278042-338271)		15.709 (14.144-17.25)	-0.58 (-1.02 to -0.14)
High SDI	475234 (449623-489570)	42.794 (40.546-44.066)		839755 (764451-885156)	40.525 (37.445-42.447)	-0.30 (-0.69 - 0.08)	243217 (227488-251652)	21.859 (20.429-22.642)		336566 (299022-359613)		15.017 (13.576-15.925)	-1.36 (-1.74 to -0.98)
GBD regions													
Andean Latin America	1902 (1648-2186)	9.492 (8.243-10.868)		8452 (6699-10526)	14.389 (11.395-17.891)	1.43 (1.32 - 1.53)	1723 (1495-1967)	8.881 (7.714-10.08)		5778 (4597-7047)		10.004 (7.975-12.183)	0.46 (0.35 - 0.57)
Australasia	11843 (10910-12804)	50.584 (46.643-54.647)		23281 (20502-26413)	43.968 (38.907-49.552)	-0.58 (-0.71 to -0.44)	5778 (5305-6246)	24.879 (22.769-26.91)		8276 (7179-9408)		14.618 (12.769-16.519)	-1.92 (-2.01 to -1.82)
Caribbean	6207 (5825-6607)	24.091 (22.575-25.673)		18481 (16074-20936)	34.332 (29.87-38.855)	1.26 (1.19 - 1.34)	3472 (3239-3721)	13.959 (13.005-14.946)		7882 (6867-8970)		14.573 (12.717-16.595)	0.27 (0.23 - 0.31)
Central Asia	6180 (5774-6592)	12.899 (12.025-13.793)		8893 (7942-9809)	10.823 (9.692-11.904)	-0.15 (-0.36 - 0.06)	4831 (4499-5157)	10.352 (9.606-11.058)		6146 (5493-6772)		7.867 (7.055-8.655)	-0.50 (-0.65 to -0.34)
Central Europe	42182 (40230-43856)	28.319 (27.029-29.454)		85867 (79290-92787)	38.817 (35.706-41.958)	0.98 (0.81 - 1.15)	32120 (30603-33396)	22.044 (21.003-22.93)		51843 (47752-55681)		22.585 (20.808-24.275)	-0.03 (-0.15 - 0.10)
Central Latin America	7632 (7311-7896)	9.325 (8.904-9.668)		44552 (39665-49721)	17.745 (15.755-19.812)	2.05 (1.99 - 2.11)	5616 (5368-5819)	7.209 (6.851-7.49)		22934 (20342-25521)		9.302 (8.259-10.351)	0.86 (0.78 - 0.94)
Central Sub-Saharan Africa	1591 (1268-1964)	7.401 (6.062-9.005)		4206 (3206-5574)	7.869 (6.094-10.535)	0.24 (0.06 - 0.41)	1494 (1200-1838)	7.426 (6.05-9.035)		3687 (2802-4932)		7.453 (5.766-10.144)	0.06 (-0.09 - 0.21)
East Asia	165083 (142142-189658)	19.081 (16.55-21.794)		684927 (559523-823301)	31.602 (25.896-37.848)	1.75 (1.65 - 1.84)	123638 (106930-141857)	15.432 (13.394-17.609)		287880 (235559-343280)		13.782 (11.325-16.346)	-0.44 (-0.50 to -0.39)
Eastern Europe	71751 (69017-73943)	25.515 (24.499-26.322)		113252 (104415-122489)	32.106 (29.592-34.73)	0.62 (0.51 - 0.74)	50097 (48178-51688)	18.062 (17.33-18.645)		64373 (59115-69822)		18.055 (16.583-19.563)	-0.19 (-0.31 to -0.07)
Eastern Sub-Saharan Africa	8179 (6323-9308)		11.078 (8.748-12.514)	17952 (15710-20779)	11.232 (9.843-12.769)	-0.10 (-0.22 - 0.01)	7779 (5998-8844)	11.058 (8.709-12.491)	15967 (13940-18320)		10.762 (9.407-12.151)		-0.21 (-0.31 to -0.12)
High-income Asia Pacific	79543 (75502-82394)		39.716 (37.427-41.173)	207277 (179498-223332)	44.89 (40.2-47.85)	0.33 (0.23 - 0.44)	35636 (33643-36954)	18.454 (17.226-19.201)	80691 (67267-88001)		14.995 (13.033-16.094)		-0.73 (-0.78 to -0.69)
High-income North America	167645 (155818-174737)		47.336 (44.189-49.256)	244681 (226550-256377)	38.75 (36.135-40.479)	-0.80 (-0.93 to -0.67)	73964 (67846-77460)	20.581 (18.952-21.519)	85865 (77871-90928)		12.955 (11.892-13.652)		-1.63 (-1.69 to -1.57)
North Africa and Middle East	17568 (14963-19790)		10.397 (9.081-11.628)	66087 (58130-74936)	14.429 (12.67-16.349)	1.31 (1.15 - 1.48)	14108 (12126-15839)	8.99 (7.935-10.061)	37395 (32759-42274)		8.945 (7.853-10.08)		0.21 (0.06 - 0.36)
Oceania	197 (162-239)		6.821 (5.749-8.029)	482 (410-560)	6.444 (5.549-7.419)	-0.18 (-0.27 to -0.09)	165 (136-200)	6.307 (5.365-7.417)	382 (324-446)		5.584 (4.798-6.416)		-0.37 (-0.47 to -0.26)
South Asia	28138 (24016-31934)		4.69 (3.997-5.343)	85115 (76614-95248)	5.647 (5.08-6.298)	0.46 (0.33 - 0.60)	25281 (21597-28741)	4.424 (3.764-5.058)	66943 (60197-74844)		4.626 (4.165-5.159)		0.02 (-0.09 - 0.13)
Southeast Asia	29321 (24900-33179)		11.278 (9.643-12.719)	116942 (101260-132256)	17.699 (15.407-19.894)	1.45 (1.40 - 1.50)	24777 (20969-28065)	10.108 (8.661-11.383)	79420 (68450-89290)		12.744 (11.115-14.264)		0.74 (0.67 - 0.80)
Southern Latin America	10983 (10130-11892)		24.09 (22.115-26.133)	24747 (21922-27561)	28.316 (25.1-31.528)	0.73 (0.56 - 0.90)	8974 (8274-9706)	20.128 (18.483-21.806)	16117 (14307-18002)		18.125 (16.116-20.247)		-0.10 (-0.27 - 0.07)
Southern Sub-Saharan Africa	2516 (2234-3057)		9.46 (8.371-11.648)	7623 (6875-8472)	13.449 (12.157-14.825)	1.29 (1.06 - 1.52)	2225 (1974-2720)	8.822 (7.792-10.938)	6130 (5550-6786)		11.465 (10.368-12.61)		0.97 (0.70 - 1.25)
Tropical Latin America	9843 (9351-10324)		11 (10.322-11.564)	44245 (40859-47144)	17.169 (15.817-18.314)	1.42 (1.31 - 1.54)	8102 (7660-8506)	9.554 (8.906-10.061)	29415 (26981-31339)		11.578 (10.589-12.342)		0.66 (0.57 - 0.75)
Western Europe	243931 (229598-253378)		41.812 (39.506-43.397)	375462 (337713-401850)	40.54 (37.17-43.067)	-0.11 (-0.26 - 0.04)	136377 (127108-142174)	22.976 (21.452-23.926)	156637 (136857-169939)		15.112 (13.529-16.253)		-1.40 (-1.45 to -1.36)
Western Sub-Saharan Africa	4347 (3709-5101)		5.198 (4.439-6.037)	11620 (9673-13673)	6.285 (5.362-7.297)	0.78 (0.71 - 0.85)	4162 (3557-4867)	5.217 (4.483-6.042)	10312 (8668-12100)		5.98 (5.121-6.882)		0.62 (0.55 - 0.69)

ASIR, Age-Standardized Incidence Rate; ASDR, Age-Standardized Death Rate; DALYs, Disability-Adjusted Life Years; EAPC, Estimated Annual Percentage Change; CRC, Colorectal Cancer; SDI, Socio-demographic Index; UI, Uncertainty Interval.

**Table 2:** The DALYs, Age-standardized DALYs rate, and EAPCs of CRC at the global, regional levels, categorized by SDI and sex from 1990 to 2021.

Characteristics	1990		2021		
	DALYs case	Age_standardized DALYs Rate	DALYs case	Age_standardized DALYs Rate	EAPC
	(95%UI)	per 100,000(95%UI)	(95%UI)	per 100,000(95%UI)	per 100,000(95%CI)
Global	14396658 (13568749-15166576)	357.326 (336.623-375.739)	24401100 (22689369-26161518)	283.242 (263.114-303.326)	-0.83 (-0.87 to -0.80)
Gender					
Female	6786955 (6292858-7304106)	316.541 (292.928-340.378)	10233851 (9257556-11064615)	224.295 (203.211-242.65)	-1.25 (-1.30 to -1.19)
Male	7609703 (7037903-8139915)	405.58 (378.494-431.931)	14167249 (12782332-15683971)	349.668 (316.681-386.643)	-0.52 (-0.55 to -0.50)
SDI regions					
Low SDI	452673 (350578-521203)	181.867 (142.036-208.318)	901645 (799642-1014005)	162.293 (145.25-181.893)	-0.48 (-0.62 to -0.35)
Low-middle SDI	1021299 (879104-1160914)	149.424 (129.02-169.501)	2564121 (2333601-2815224)	166.475 (151.967-182.499)	0.38 (0.33 - 0.43)
Middle SDI	3142818 (2820037-3490094)	273.628 (246.603-302.131)	7120502 (6308564-7920507)	259.354 (230.172-288.242)	-0.28 (-0.63 - 0.08)
High-middle SDI	4416013 (4153232-4663023)	436.817 (410.011-460.375)	7079678 (6397925-7847048)	364.611 (329.264-404.767)	-0.75 (-1.18 to -0.32)
High SDI	5344611 (5120390-5504892)	490.502 (470.409-505.012)	6705749 (6179256-7070831)	338.225 (316.751-354.913)	-1.33 (-1.72 to -0.94)
GBD regions					
Andean Latin America	43988 (37838-50561)	203.465 (175.153-233.97)	136183 (108535-167982)	226.761 (180.593-279.799)	0.39 (0.27 - 0.50)
Australasia	133623 (123838-143847)	577.928 (535.913-621.912)	166740 (147524-186891)	327.189 (290.904-364.679)	-2.06 (-2.17 to -1.96)
Caribbean	83680 (78049-89689)	317.263 (295.898-339.948)	179752 (155874-205420)	335.019 (290.413-383.111)	0.33 (0.28 - 0.37)
Central Asia	140374 (132361-149146)	280.689 (263.918-298.738)	169952 (151537-187708)	196.551 (175.594-216.681)	-0.88 (-1.00 to -0.76)
Central Europe	767091 (735174-795936)	512.391 (489.921-531.482)	1087562 (1003626-1168378)	506.482 (467.975-544.568)	-0.12 (-0.25 - 0.00)
Central Latin America	149275 (143941-154015)	165.762 (159.241-171.332)	594117 (529274-662367)	231.951 (206.641-258.569)	1.11 (1.03 - 1.18)
Central Sub-Saharan Africa	44003 (35103-54650)	179.378 (145.644-220.18)	109622 (82688-146971)	177.792 (135.169-238.587)	0.02 (-0.12 - 0.17)
East Asia	3691552 (3152648-4246182)	389.57 (334.251-446.899)	7148995 (5822935-8561079)	334.505 (274.007-399.929)	-0.59 (-0.66 to -0.51)
Eastern Europe	1280866 (1237775-1320907)	455.739 (440.174-469.529)	1465104 (1343981-1600585)	424.543 (389.695-463.673)	-0.48 (-0.61 to -0.35)
Eastern Sub-Saharan Africa	225496 (171119-258759)	274.478 (211.05-312.214)	444253 (385800-525458)	242.129 (211.299-279.078)	-0.58 (-0.68 to -0.47)
High-income Asia Pacific	874132 (837041-905135)	431.838 (411.772-447.547)	1441630 (1272496-1549759)	331.482 (303.848-352.473)	-0.93 (-0.98 to -0.88)
High-income North America	1613202 (1529313-1679101)	469.381 (446.298-487.526)	1907928 (1788111-2003824)	316.043 (298.523-330.84)	-1.36 (-1.42 to -1.31)
North Africa and Middle East	409788 (341302-464924)	220.981 (187.753-248.961)	1012652 (886199-1154503)	209.038 (183.159-237.276)	-0.01 (-0.15 - 0.12)
Oceania	5153 (4142-6362)	154.389 (127.38-186.626)	11643 (9764-13750)	136.647 (116.102-159.447)	-0.39 (-0.48 to -0.29)
South Asia	787251 (675898-889380)	119.116 (101.907-135.156)	1908668 (1711982-2155055)	120.375 (108.304-135.299)	-0.10 (-0.21 - 0.01)
Southeast Asia	735822 (612422-841712)	257.932 (218.13-292.166)	2166650 (1868880-2456350)	313.372 (270.746-353.54)	0.61 (0.55 - 0.67)
Southern Latin America	206237 (189818-224031)	445.856 (410.176-484.75)	349581 (311369-391893)	407.99 (363.051-457.564)	-0.04 (-0.19 - 0.11)
Southern Sub-Saharan Africa	60914 (54662-72328)	207.844 (185.416-251.149)	166062 (149959-186753)	270.635 (244.941-301.851)	1.06 (0.78 - 1.35)
Tropical Latin America	218357 (209209-228658)	224.277 (213.275-235.189)	744957 (694956-786849)	286.216 (266.669-302.446)	0.77 (0.67 - 0.87)
Western Europe	2815472 (2680130-2917395)	498.566 (476.346-516.244)	2912355 (2641021-3112519)	326.815 (301.667-347.226)	-1.41 (-1.46 to -1.36)
Western Sub-Saharan Africa	110381 (93741-130867)	120.111 (102.578-141.263)	276692 (224568-328426)	132.051 (110.39-155.48)	0.46 (0.40 - 0.52)

DALYs, Disability-Adjusted Life Years; EAPC, Estimated Annual Percentage Change; CRC, Colorectal Cancer; SDI, Socio-demographic Index; UI, Uncertainty Interval.

**Figure 2:**
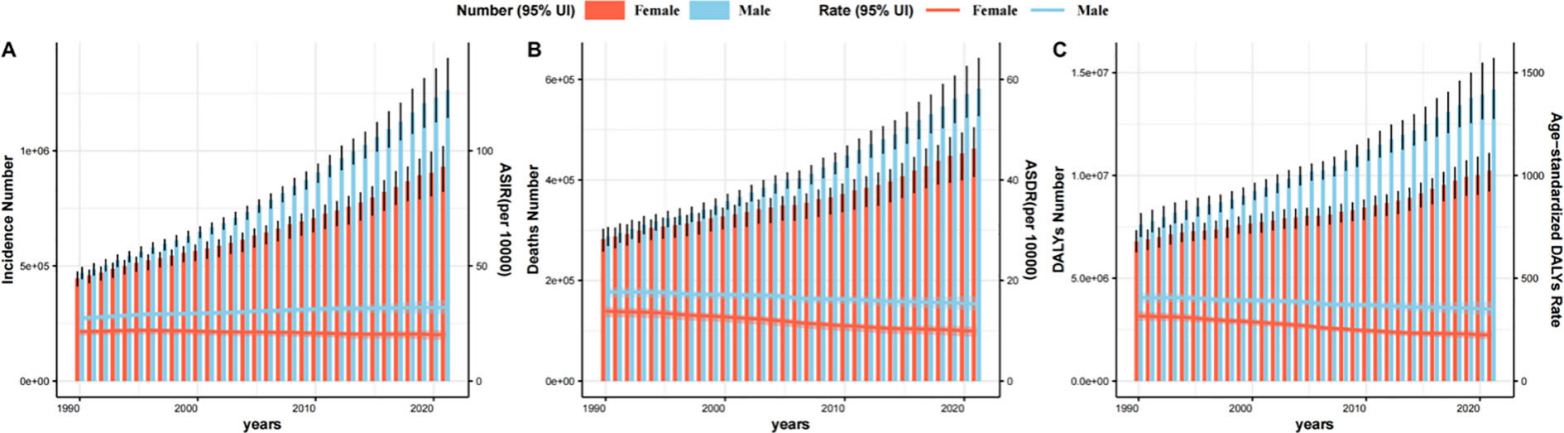
Global trends from 1990 to 2021 in number and crude rate of incidence**(A)**, deaths **(B)** and DALYs **(C)** of CRC by sex. ASIR, Age-Standardized Incidence Rate; ASDR, Age-Standardized Death Rate; DALYs, Disability-Adjusted Life Years; CRC, Colorectal Cancer.

In 2021, the global ASDR was 12.398 (95% UI: 11.241–13.306), and the age-standardized DALYs rate was 283.242 (263.114-303.326) (**Tables 1**, **2**). Among regions, Central Europe had the highest ASDR at 22.585 (95% UI: 20.808–24.275), followed by Southern Latin America at 18.125 (95% UI: 16.116–20.247) and Eastern Europe at 18.055 (95% UI: 16.583–19.563). At the national level, Uruguay recorded the highest overall ASDR at 27.461 (95% UI: 24.253–30.906), followed by Hungary at 26.007 (95% UI: 21.735–31.128) and Bulgaria at 25.713 (95% UI: 20.964–30.734) (**Figure 1**). Notably, the top three rankings for age-standardized DALYs rates at the national and regional levels mirrored those of ASDR, albeit with slight differences in order (**Figure 1**). The EAPC for ASDR was -0.81 (95% CI: -0.84 to -0.77), while the EAPC for the age-standardized DALYs rate was -0.83 (95% CI: -0.87 to -0.80), indicating a significant declining trend for both metrics from 1990 to 2021. In summary, during the period from 1990 to 2021, the global trends of CRC incidence and disease burden displayed a complex pattern: on the one hand, incidence rates showed an increase; on the other hand, CRC mortality rates and age-standardized DALYs rates demonstrated a gradual decline (**Tables 1**, **2**, **Figure 2**).

### Variation in CRC burden in two sexes and five-year age groups

**Figure 3:**
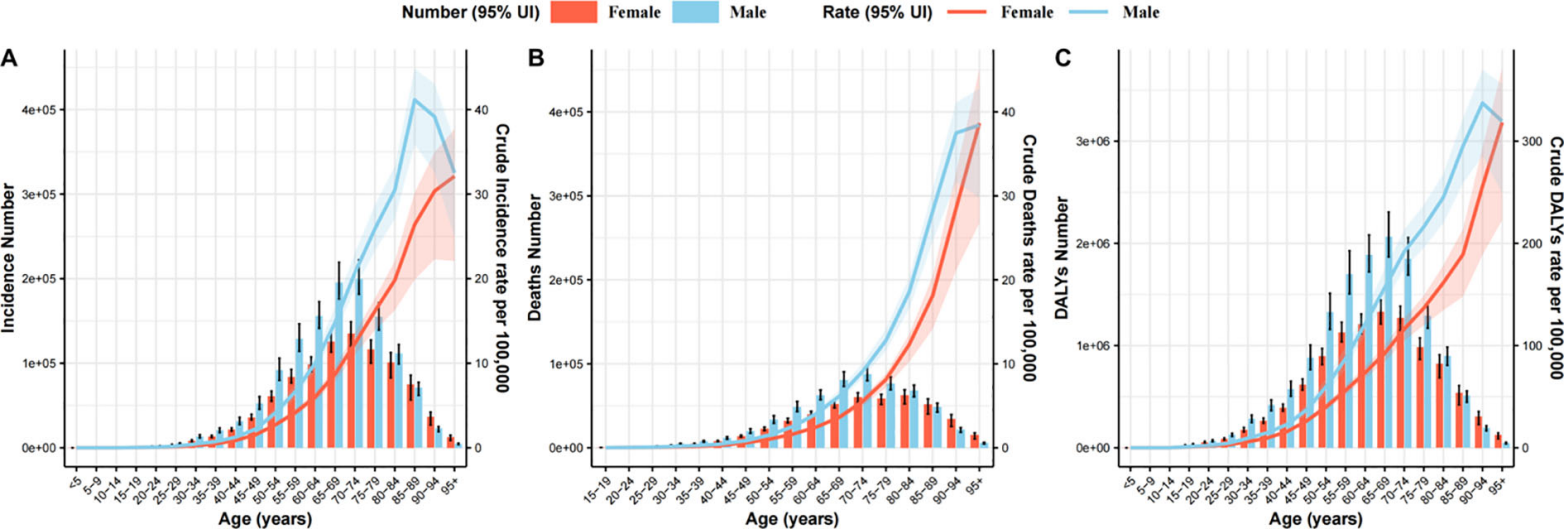
Global age patterns by sex in 2021 of number and crude rate of incidence **(A)**, deaths **(B)**, and DALYs **(C)**. Error bars indicate the 95% UI for the number or crude rates. ASIR, Age-Standardized Incidence Rate, ASDR, Age-Standardized Death Rate; DALYs, Disability-Adjusted Life Years; CRC, Colorectal Cancer; UI, Uncertainty interval.

**Figures 2** and **3** illustrate the age- and sex-specific patterns of the global CRC burden. From a gender perspective, in 2021, the global CRC incident cases, deaths, and DALYs were higher in males compared to females. Similarly, males exhibited higher ASIR, ASDR, and age-standardized DALYs rates than females. From an age-specific perspective, in 2021, the DALYs were highest in the 65–69 age group for both men and women, while the number of incident cases and deaths peaked in the 70–74 age group. Among females, the highest crude incidence rate, crude mortality rate, and crude DALYs rate were observed in those aged 95+, whereas in males, these peak values occurred in the 85–89 and 90–94 age groups, respectively (**Figure 3**).

From 1990 to 2021, the incidence cases, mortality cases, and DALYs for both sexes consistently increased, with males exhibiting higher values than females each year. The ASDR and age-standardized DALYs rate in males showed a decline, with EAPCs of -0.50 (95% CI: -0.52 to -0.47) and -0.52 (95% CI: -0.55 to -0.50), respectively (**Figure 2**). In females, the decline was more pronounced, with EAPCs of -1.19 (95% CI: -1.24 to -1.14) and -1.25 (95% CI: -1.30 to -1.19). Notably, between 1990 and 2021, only males exhibited an upward trend in ASIR, with an EAPC of 0.45 (95% CI: 0.50 to 0.54), whereas females showed a steady decline, with an EAPC of -0.34 (95% CI: -0.29 to -0.25) (**Table 1**).

### Variation in CRC burden by SDI

**Figure 4:**
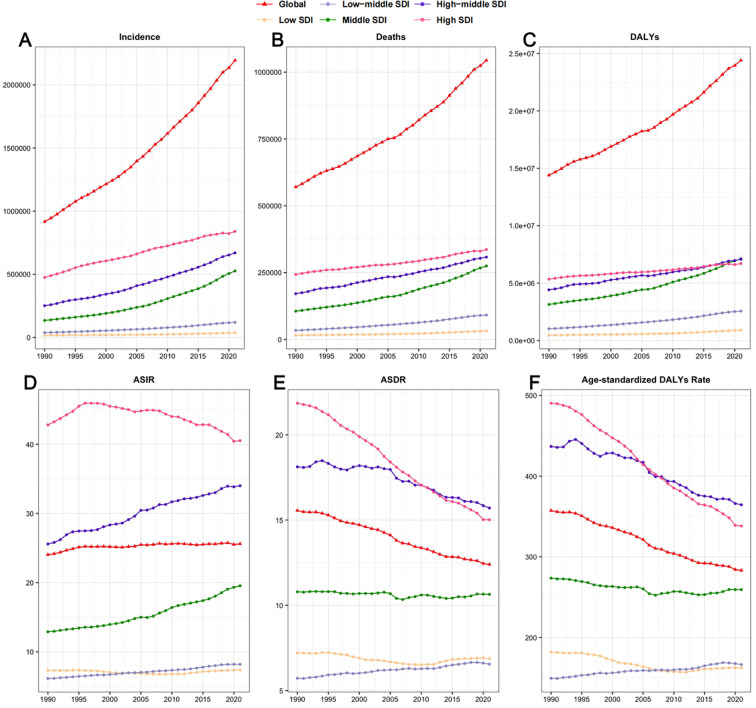
Trends in the incident cases **(A)**, deaths **(B)**, DALYs **(C)**, ASIR **(D)**, ASDR **(E)** and Age-standardized DALYs rate **(F)** of CRC from 1990 to 2021 by different SDI level regions. ASIR, Age-Standardized Incidence Rate; ASDR, Age-Standardized Death Rate; DALYs, Disability-Adjusted Life Years; CRC, Colorectal Cancer; SDI, Socio-demographic Index.

**Figure 5:**
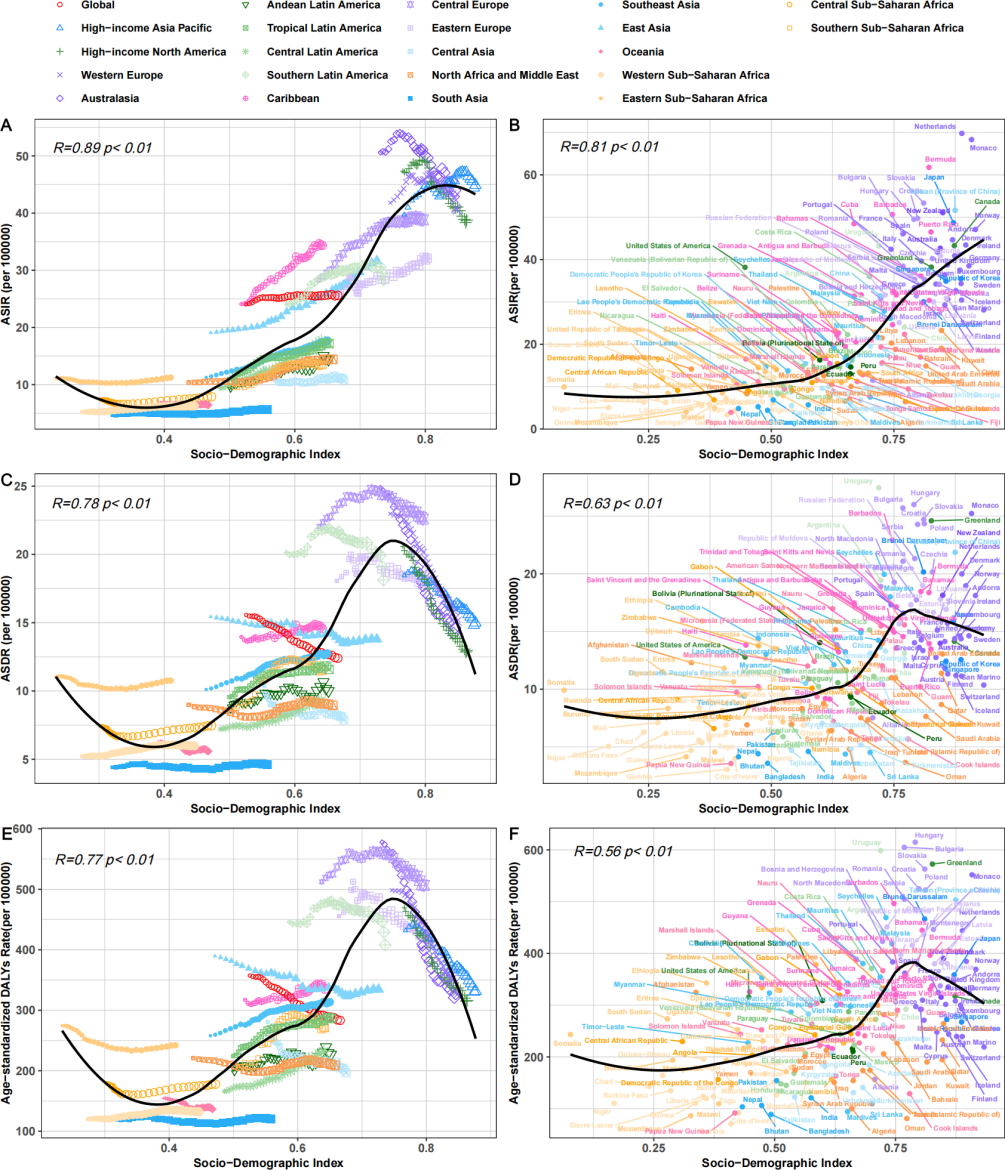
The correlation between the SDI and the ASIR **(A, B)**, ASDR **(C, D)**, and Age-standardized DALYs rate **(E, F)** of CRC per 100,000 population across 21 GBD regions and 204 countries globally. SDI, Socio-demographic Index; ASIR, Age-Standardized Incidence Rate; ASDR, Age-Standardized Death Rate; DALYs, Disability-Adjusted Life Years; CRC, Colorectal Cancer.

We further explored the differences between these regions using the SDI. **Figures 4** and **5** clearly reveal a distinct socioeconomic gradient in the CRC burden. From 1990 to 2021, the incident cases, deaths, and DALYs of CRC increased across all SDI regions, which is consistent with the findings from 2019 (**Figure 4**) (17). The ASIR in high and high-middle SDI regions was higher than in other regions, and a positive correlation was observed between ASIR and SDI across 204 countries (R = 0.81, p< 0.05) (**Figure 5**).

In high-SDI regions, the ASIR of 40.525 (37.445–42.447) was significantly higher than in other regions. Despite the persistently high incidence rates, the ASDR of 15.017 (13.576–15.925) and the age-standardized DALYs rate of 338.225 (316.751–354.913) showed a notable declining trend. In contrast, middle-high SDI regions recorded the highest ASDR of 15.709 (14.144–17.25) and age-standardized DALYs rate of 364.611 (329.264–404.767). In middle-low SDI regions, the EAPC for ASDR was 0.48 (0.43–0.54), and for the age-standardized DALYs rate, it was 0.38 (0.33–0.43), the highest among all SDI regions. This indicates that while middle-high SDI regions bear a heavier burden of CRC-related deaths and disabilities, the burden in middle-low SDI regions is increasing at a faster pace (**Tables 1**, **2**, **Figure 4**). This reveals the varying impact of CRC across regions with different levels of development globally.

### Prediction of CRC ASIR, ASDR and age-standardized DALYs rate in the next decade

**Figure 6:**
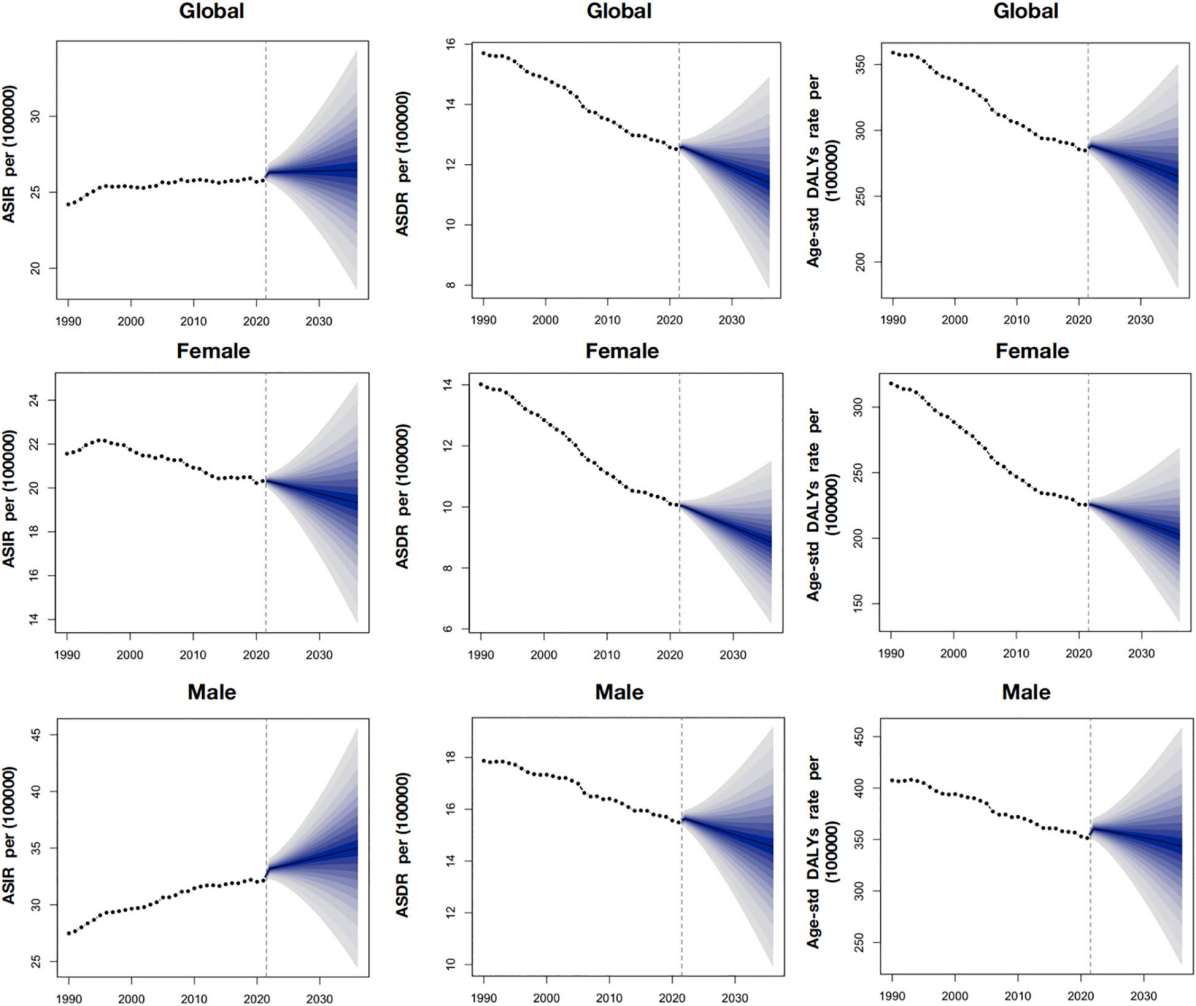
Global Forecasts of CRC ASIR, ASDR, and Age-standardized DALYs rate from 1990 to 2035 by BAPC. ASIR, Age-Standardized Incidence Rate; ASDR, Age-Standardized Death Rate; DALYs, Disability-Adjusted Life Years; CRC, Colorectal Cancer; BAPC, Bayesian Age-Period-Cohort.

**Figure 6** presents the projections from our BAPC model. In this study, we utilized the GBD data for CRC from 1990 to 2021 and employed the BAPC to predict the disease burden trend of CRC from 2022 to 2036 based on age groups (**Figure 6**). The results show that over the next 15 years, the global CRC ASIR will experience a slow and moderate upward trend, expected to increase from 25.6 per 100,000 in 2021 to 26.5 per 100,000 in 2036. However, both ASDR and age-standardized DALYs are expected to continue to decline significantly in the coming 15 years. It is estimated that the ASDR will decrease from 12.4 per 100,000 in 2021 to 11.4 per 100,000 in 2036, while the age-standardized DALYs rate will drop from 283.2 per 100,000 in 2021 to 265.1 per 100000. From a gender-specific perspective, between 2022 and 2026, the global CRC ASIR will show a decreasing trend for females and an increasing trend for males (with females expected to decrease from 20.2 per 100,000 to 19.3 per 100,000 and males expected to increase from 31.9 per 100,000 to 35 per 100,000). In contrast, both ASDR and age-standardized DALYs rates for all genders are expected to maintain a continuous downward trend.

## Discussion

In 2016, the World Health Organization introduced the *2030 Agenda for Sustainable Development*, aiming to strengthen the governance, management, and monitoring of non-communicable diseases (NCDs). The agenda outlines the goal to “ reduce premature deaths from NCDs by one third by 2030,” highlighting that this was an issue that “the issue that the Millennium Development Goals did not address.” (18) CRC patients are closely related to this goal, as CRC is currently the second leading cause of cancer-related deaths globally, with its mortality rate ranking only after lung cancer. On average, 13 out of every 100,000 people die from CRC, accounting for 1.41% of the global deaths in 2021. Therefore, comprehensively understanding the trends of CRC prevalence is crucial for assessing the potential to achieve related health goals. However, there is currently a lack of comprehensive literature analysis on the incidence, mortality, and DALYs of CRC. Based on this, we analyze the temporal development trends of CRC disease burden using GBD data and assess it by region, country, age, and gender. This approach helps decision-makers understand the CRC burden in specific regions and populations and develop effective prevention and control strategies, thereby promoting the effective management and prevention of CRC disease burden.

From 1990 to 2021, the global incident cases, deaths, and DALYs related to CRC significantly increased. Additionally, the global ASIR of CRC increased, while both ASDR and age-standardized DALYs rates showed a decreasing trend. This result indicates that while the incidence of new CRC cases is rising, the burden on patients is decreasing, a divergence in trends that is in line with observations from other screening-detectable cancers (19). The reasons for this may include improvements in tumor patient information registration, as well as significant progress in early diagnosis, screening, and patient care quality. The widespread adoption of CRC screening is one of the key mechanisms driving the increase in ASIR. However, during the initial and intermediate phases of screening implementation, the detection of many previously undiagnosed early-stage cases and precancerous lesions temporarily elevates the incidence rate—a phenomenon known as the “detection effect” (20–22). Additionally, advances in CRC surgical techniques, the increase in cancer-directed surgeries, progress in liver metastasis treatment, and the development and optimization of targeted therapies have greatly improved the 5-year survival rate for CRC patients, and survival time for those with advanced disease has also started to extend, thereby leading to a reduction in both ASDR and DALYs for CRC (20, 23).

In 2021, the ASIR, ASDR, and age-standardized DALYs rates for CRC were highest in high and high-middle SDI regions. Moreover, over the past 32 years, the decline in CRC disease burden has been most notable in high SDI regions. In general, SDI reflects the economic and social development level of a country or region. Populations in highly developed countries or regions are more likely to have sedentary lifestyles, consume more red meat and highly processed foods, smoke more cigarettes, drink more alcohol, and have higher rates of obesity and diabetes, all of which are considered risk factors for CRC (1, 24, 25). Additionally, a higher SDI level also signifies a more robust healthcare system and higher quality medical services, particularly the rapid spread and development of colonoscopy screening. In this study, the most significant increase in ASIR, ASDR, and age-standardized DALYs rates was observed in regions with moderate and low-middle SDI, which aligns with conclusions from previous research on the evolution of cancer burden (4). The most reasonable hypothesis for this result is that, with the economic development of mid-SDI regions, rapid urbanization and industrialization have altered people’s lifestyles, leading to an increase in CRC risk factors and disease diagnoses. These factors include environmental pollution (26), food safety issues (27), lack of physical activity (28), overuse of medications and electronic devices, and deteriorating sleep patterns (29). Additionally, these regions often face constraints in healthcare infrastructure, including inadequate screening programs, diagnostic delays, and limited access to advanced treatments, collectively contributing to a higher disease burden (30, 31). It is imperative to establish a cost-effective, stepwise screening system. In resource-limited areas, priority should be given to promoting primary screening methods such as fecal immunochemical testing (FIT), followed by confirmatory colonoscopy for positive cases, while enhancing the diagnostic and treatment capabilities of primary healthcare workers through targeted training.

Between 1990 and 2021, the regions of Central Latin America and Sub-Saharan Africa, particularly the southern parts, showed the most significant increases in CRC ASIR, ASDR, and age-standardized DALYs rates. This could be attributed to the scarcity of healthcare services and environmental pollution resulting from rapid urbanization (20, 32). The increasing disease burden of CRC in economically burdened and resource-scarce regions is particularly concerning. Many countries in middle- and low-SDI regions lack adequate access to CRC prevention services, timely diagnosis, and comprehensive treatment. Therefore, strengthening healthcare infrastructure, expanding medical workforce capacity, and improving universal health coverage and financial security are crucial (4). Considering the economic resources and healthcare infrastructure in these regions, relatively inexpensive screening measures, such as fecal occult blood testing, may be a feasible strategy to alleviate the CRC burden (5). In addition, we found significant differences in CRC’s ASIR, ASDR, and age-standardized DALYs rates among different countries within the same region, exhibiting a clear polarization trend. For example, Hungary and Albania, both located in Central Europe and having similar SDI levels, show a disparity of up to three times in CRC disease burden. This difference may be attributed to varying national policies regarding medical technologies and healthcare for CRC prevention. Therefore, continuous monitoring of CRC-related indicators in these countries can provide valuable insights and references for the development of global CRC prevention and control strategies.

Our study suggests that the burden of CRC in males is higher, and the growth rate of the burden is faster compared to females. In the next 15 years, the ASDR and age-standardized DALYs rates for female CRC patients are expected to decrease more significantly than in males, while the ASIR for male CRC patients will continue to rise and remain at a high level. For males, unhealthy diet, smoking, alcohol consumption, and high BMI have long been associated with CRC incidence and prognosis, with a stronger correlation between high BMI and CRC risk in males (33). In addition, heme iron, which contributes to the production of reactive oxygen species (ROS) and alters the gut microbiota, is also involved in the development of CRC. Since iron deficiency is less common in males, this may also contribute to the higher incidence of CRC in males compared to females (1). From an age-specific perspective, in 2021, the highest crude incidence and crude mortality rates for CRC were observed in the >85 age group. This may be attributed to the global aging population and the higher prevalence of CRC-related complications among elderly patients. It highlights the importance of special attention and health management for this aging population (34). It is noteworthy that the highest peak in female CRC incidence occurs at an older age (95+) compared to males (85-89). This phenomenon may be the result of a combination of biological sex differences and attitudes toward seeking healthcare. Therefore, targeted interventions are necessary, and for men, widespread education on CRC prevention and control is particularly important.

This study has several methodological and data-related limitations. First, although the GBD data used in the analysis offer broad coverage and comparability, their quality and completeness directly affect the accuracy of the results. Moreover, in some countries and regions—particularly those with underdeveloped health information systems—actual disease burden data are often missing, and GBD relies on modeling methods for estimation, which may introduce uncertainty. Third, differences in data collection and coding practices across countries in the GBD study, despite standardization efforts, may still lead to some degree of data bias. Fourth, although the ecological analysis approach adopted in this study can reveal macro-level trends and associations, it cannot infer causality at the individual level, nor can it fully control for confounding factors. Despite these limitations, our findings, based on large-sample cancer data and advanced modeling methods, still clearly illustrate the evolving trends of colorectal cancer burden in the past, present, and future.

## Conclusions

Overall, our ecological analysis of GBD 2021 data shows a gradual increase in the global CRC ASIR against notable declines in both ASDR and age-standardized DALYs rate. This trend aligns with previous GBD studies (35), while our findings provide novel insights: the disease burden exhibits a clear negative correlation with SDI, characterized by the most pronounced increases in middle-SDI regions and significant disparities between countries. We identify men and older adults (>85 years) as high-risk groups. These results underscore the urgent need for targeted, international strategies that integrate socioeconomic, gender, and aging factors to manage the global CRC burden effectively.

## Data Availability

The original contributions presented in the study are included in the article/supplementary material. Further inquiries can be directed to the corresponding author.
